# Factors Associated with Extreme Waiting Times for Child Developmental Diagnostic Assessments: A Mixed-Methods Service Evaluation in Sydney

**DOI:** 10.3390/children13081088

**Published:** 2026-08-17

**Authors:** Jasmine Rosewarne, Pankaj Garg, Romy Hurwitz, Sinthu Vivekanandarajah, Shanti Raman

**Affiliations:** 1 Department of Community Paediatrics, South Western Sydney Local Health District, Sydney, NSW 2170, Australia; jasmine.rosewarne@health.nsw.gov.au (J.R.); romy.hurwitz@health.nsw.gov.au (R.H.); sinthu.vivekanandarajah@health.nsw.gov.au (S.V.); shanti.raman@health.nsw.gov.au (S.R.); 2 Discipline of Paediatrics and Child Health, UNSW Medicine, The University of New South Wales (UNSW Sydney), Sydney, NSW 2052, Australia; 3 Discipline of Paediatrics and Child Health, Western Sydney University, Richmond, NSW 2753, Australia; 4 School of Population Health, The University of New South Wales (UNSW Sydney), Sydney, NSW 2170, Australia; 5 Child and Family Health, Healthy Families and Communities Branch, New South Wales Department of Health, North Sydney, NSW 2065, Australia

**Keywords:** developmental assessment, waiting times, healthcare access, mixed methods, service evaluation

## Abstract

**Highlights:**

**What are the main findings?**

• Validation of administrative waiting-time data showed that almost one-third of records initially identified as extreme waits were misclassified because of administrative inaccuracies.
• Children experiencing extreme waiting times demonstrated distinct demographic and service characteristics compared with the overall child developmental assessment service cohort.
• Caregivers described extreme waiting as being characterised by communication challenges, uncertainty regarding service pathways, reliance on interim supports, and financial barriers to private assessment.

**What are the implications?**

• Validation of administrative data is essential before waiting-time metrics are used for service evaluation or planning.
• Improved waitlist communication, service navigation, and equitable access to interim supports may improve caregiver experiences while children await developmental assessments.
• Identifying client and service characteristics associated with extreme waiting may support more equitable prioritisation and future service redesign.

**Abstract:**

**Background:** Previous Australian child developmental assessment service (CDAS) cohorts have identified a small proportion of children experiencing extreme waiting times (≥730 days) for standardised developmental assessments. However, factors associated with these extreme waits remain poorly understood. **Aim:** To examine factors associated with a priori defined extreme waiting times (≥730 days) by comparing affected children with the remaining CDAS cohort, while validating administrative data and exploring caregiver experiences. **Methods:** An embedded mixed-methods service evaluation was undertaken. Quantitative data were extracted from the CDAS database and electronic medical records for children assessed between October 2022 and September 2024. Children with validated extreme waiting times were compared with the remaining CDAS cohort. Records initially identified as waiting ≥730 days underwent manual validation of referral and assessment dates. Caregivers of children with validated extreme waits were purposively recruited for semi-structured interviews. Fifteen caregivers participated; twelve families did not respond. Interview transcripts underwent thematic analysis. **Results:** Of 67 children initially identified as waiting ≥730 days, 20 (29.9%) were excluded following manual validation because of administrative recording inaccuracies, leaving 47 children with confirmed extreme waiting times. Of these, 46 completed CDAS assessment and had available validated waiting-time data, with a median wait of 859 days (IQR 775–932), compared with 254 days (IQR 111–355) in the comparison cohort of 2039 other children. Children in the extreme-wait cohort were more often from culturally and linguistically diverse backgrounds (76.6% vs. 53.5%; *p* = 0.002), required interpreters more frequently (23.4% vs. 9.0%; *p* = 0.003), and were more likely to be referred by paediatricians (89.4% vs. 74.1%; *p* = 0.017). Over one-third (*n* = 17) were prioritised for assessment within six months at triage. Review assessments accounted for 25/47 (53.2%) of the extreme-wait cohort. Caregivers described acceptance of lengthy public-sector waits, communication gaps, uncertainty regarding service pathways, reliance on interim supports, financial barriers to private assessment, and positive experiences once assessments were completed. **Conclusions:** Children experiencing extreme waits had distinct characteristics. Extreme waiting reflected genuine service delays, planned clinical review pathways, and administrative recording inaccuracies. Administrative data should be validated before waiting-time metrics are used for service evaluation. Improved communication, service navigation, equitable access to interim supports, and targeted service redesign may improve caregiver experiences and reduce extreme waits in publicly funded developmental assessment services.

## 1. Introduction

Timely access to standardised developmental assessments is essential for diagnostic classification, early intervention and appropriate care planning for children with developmental concerns [1,2,3]. However, prolonged waiting times for developmental assessments are widely reported internationally, creating barriers to timely diagnosis and intervention and highlighting the need for robust surveillance of waiting times within child developmental assessment services (CDASs) [2,4,5].

To address this, CDAS services in Sydney, Australia developed standardised data collection and triage systems to monitor service performance and equity of access [6,7]. Previous Australian evaluations using these systems reported a median waiting time of approximately 10 months (302 days), demonstrated improved access for culturally and linguistically diverse (CALD) families, and informed the development of targeted pre-school assessment clinics [6,8]. These studies also identified a small group of children experiencing extreme waiting times of ≥730 days (2 years) for developmental assessment.

Although studies from the United Kingdom, Canada and other high-income countries report developmental and autism diagnostic waiting times commonly exceeding 12–18 months, little is known about children experiencing extreme waiting times (≥730 days), particularly within Australian contexts [9,10,11,12]. Understanding this cohort is important because extreme delays may result in missed opportunities for intervention, increased caregiver stress and widening developmental and educational disparities with the potential to reinforce vulnerabilities [9,10].

Extreme waiting times are likely multifactorial in aetiology. Contributing factors include increasing referral numbers, workforce shortages, administrative processes, prioritisation systems and the complexity of developmental assessments with limited multidisciplinary capacity [2,4,5,11]. For example, Parr et al. reported a 115% increase in referrals to autism diagnostic services between 2015 and 2019 [2]. However, Australian evidence suggests that not all long waits necessarily represent service failure. Some children may appropriately receive lower assessment priority because they already have access to early intervention services, have well-supported families, or clinicians consider that intervention should commence before formal diagnostic assessment [6]. Consequently, reported waiting times may reflect a combination of genuine service delays, planned clinical pathways and administrative recording practices. Despite their importance for service evaluation and resource allocation, few studies have examined these contributors or validated the accuracy of routinely collected administrative waiting-time data.

In addition to service factors, children from CALD backgrounds, refugee families and socioeconomically disadvantaged communities may experience additional barriers when accessing developmental assessment services, including difficulty navigating referral pathways, uncertainty regarding waiting times, limited resources for advocacy and reduced access to interim supports [7,9,11,13,14,15].

Qualitative Australian studies have also shown that some culturally diverse families do not necessarily perceive long waiting times negatively, suggesting that acceptable waiting times may differ according to clinical circumstances and family expectations [13].

This study was conducted within the South Western Sydney Local Health District (SWSLHD), one of Australia’s largest and most culturally diverse health districts, serving more than one million residents across seven local government areas [16]. The district has high levels of cultural and linguistic diversity, refugee settlement, socioeconomic disadvantage and developmental vulnerability, creating substantial demand for equitable public developmental assessment services [16,17,18].

Previous CDAS evaluations identified a small cohort of children experiencing extreme waiting times (≥730 days), but the characteristics of these children, the contribution of planned review pathways, the accuracy of administrative waiting-time data and caregiver experiences remain poorly understood [6].

This mixed-methods service evaluation aimed to identify demographic and clinical factors associated with extreme waiting times (≥730 days), compare children experiencing extreme waits with the remaining CDAS cohort, validate administrative waiting-time data, and explore caregiver experiences to identify opportunities for service improvement.

## 2. Methodological Approach

### 2.1. Study Design

This embedded mixed-methods service evaluation combined quantitative analysis of administrative waiting-time data with qualitative exploration of caregiver experiences of extreme waiting for developmental assessment [19]. A convergent interpretation of both data sources was undertaken to provide a comprehensive understanding of extreme waiting times within the CDAS.

The quantitative component comprised a retrospective cohort study using routinely collected administrative and clinical data, while the qualitative component comprised semi-structured caregiver interviews analysed using thematic analysis. Reporting followed the Good Reporting of A Mixed Methods Study (GRAMMS) recommendations (Appendix A) and Consolidated Criteria for Reporting Qualitative Research (COREQ) for the qualitative component (Appendix D) [19].

This project was approved as a quality improvement and service evaluation activity by the South Western Sydney Local Health District Quality Improvement Committee (Reference: PCH01/2025/0). Individual consent was not required for the retrospective quantitative component because only de-identified routinely collected clinical data were analysed. Verbal informed consent was obtained from all participants before qualitative interviews and documented contemporaneously by the interviewer in accordance with the approved quality improvement protocol.

### 2.2. Study Setting

This local health district in Sydney, Australia is highly culturally and linguistically diverse: approximately 39% of residents were born overseas and 46% spoke a language other than English at home in 2021. Major overseas-born communities include people from Vietnam, Iraq, and Lebanon while Arabic and Vietnamese are among the most frequently spoken community languages. Approximately 10% of residents from non-English-speaking backgrounds report limited English proficiency. SWSLHD is also a major refugee-settlement region, predominantly in Fairfield and Liverpool [16]. Substantial socioeconomic disadvantage is concentrated within the metropolitan portion of the District as measured by Socio-Economic Indexes for Areas (SEIFA) [17]. Similarly, according to the Australian Early Development Census (AEDC), the proportion of children developmentally vulnerable on two or more domains in several service locations exceeded the New South Wales average of approximately 9.6% (Table 1) [18]. The service provides multidisciplinary developmental assessments at multiple sites to facilitate easier access for families (Table 1).

Between 2022 and 2024, CDAS completed 900–1100 developmental assessments annually while the referrals increased to about 1200–1400 referrals in exceeding service capacity. A standardised Model of Care for triage, prioritisation and data collection was implemented by the service in 2018 (Appendix B). Children underwent one of three pathways: (1) **new assessment**, the first comprehensive developmental assessment following referral; (2) **planned review assessment**, a clinician-scheduled reassessment to monitor progress or clarify diagnosis after intervention or maturation; and (3) **unplanned review assessment**, a reassessment following discharge for a new clinical concern or referral question.

### 2.3. Participants

#### 2.3.1. Quantitative Component

The CDAS database contained 2231 assessment records between October 2022 and September 2024. Following data cleaning to create a unique assessment-level dataset, two cohorts were identified: an extreme-wait cohort (≥730 days; *n* = 67) and a comparison cohort (<730 days). A total of 125 records (103 failed-to-attend appointments and 22 duplicates) were excluded, leaving 2039 unique records in the comparison cohort. Children who failed to attend did not differ significantly from the study cohort in demographic, vulnerability, referral or triage characteristics.

#### 2.3.2. Data Variables

Demographic variables included age at referral, sex, ethnicity, country of birth, culturally and linguistically diverse (CALD) status, interpreter requirement, refugee background, child protection involvement, and socioeconomic disadvantage, measured using the Australian Bureau of Statistics Socio-Economic Indexes for Areas Index of Relative Socio-Economic Disadvantage (SEIFA IRSD) [17].

CALD status was defined according to NSW Health and Australian Bureau of Statistics reporting practices [20]. Interpreter requirement indicated documented need for an accredited interpreter, while refugee background was identified from medical records.

Clinical variables included referral source, appointment pathway, triage priority, referral date, and assessment date. Triage priority followed the CDAS Model of Care, with recommended assessment timeframes of 3, 6, and 12 months for Priority 1, 2, and 3 referrals, respectively (Appendix B).

#### 2.3.3. Data Validation

Because waiting times were derived from routinely collected administrative data, all records initially identified as extreme waits underwent manual validation. Referral dates, assessment dates and appointment pathways were verified against original referrals, electronic medical records, clinic reports and appointment histories. Common discrepancies included incorrect referral dates and use of original rather than review referral dates for planned assessments. This process distinguished genuine extreme waits from administrative errors and estimated the proportion of false-positive extreme waiting records.

#### 2.3.4. Outcome Measures

The primary outcome was extreme waiting time, defined a priori as ≥730 days from referral to assessment. Secondary outcomes included demographic and clinical factors, administrative contributors, caregiver experiences, and opportunities for service improvement.

#### 2.3.5. Qualitative Data

Parents or primary caregivers of children with validated extreme waiting times were invited to semi-structured interviews. Purposive maximum-variation sampling captured diverse experiences across assessment pathway, local government area, cultural background, child age and waiting time [21].

Twenty-seven families were approached; 15 consented and completed interviews, while 12 did not respond after two contact attempts. Interviews were conducted by the first author, who had no prior involvement with the CDAS or the clinical care of most participants. Telephone interviews, guided by a semi-structured schedule (Appendix C), lasted 10–15 min, were audio-recorded with consent, transcribed verbatim, and checked for accuracy. One interview was conducted with an Arabic interpreter.

#### 2.3.6. Qualitative Data Analysis

Interview transcripts were analysed using Braun and Clarke’s six-step thematic analysis framework, which involved analysing data by familiarisation, initial coding, searching for themes, reviewing themes, defining themes and reporting findings [22].

Coding was undertaken independently by the first two authors, with discrepancies resolved by consensus. Recruitment ceased once thematic saturation was achieved. Researcher reflexivity was maintained throughout the analysis, and interpretations were reviewed collaboratively to minimise potential bias arising from one investigator’s clinical role within the CDAS. The interviewer was a female paediatric clinician (with no clinical role in CDAS), and no other person was present during interviews. No repeat interviews were conducted. Coding was undertaken manually without dedicated qualitative-analysis software. Transcripts were not routinely returned to all participants for comment or correction, and member checking was not undertaken for all participants. The qualitative component was reported in accordance with the Consolidated Criteria for Reporting Qualitative Research (COREQ) 32-item checklist (Appendix D) [23].

### 2.4. Statistical Analysis

Continuous variables were summarised as median (IQR) and categorical variables as frequencies and percentages. Demographic and clinical characteristics were compared using the Mann–Whitney U or Kruskal–Wallis test for continuous variables and Pearson’s chi-square or Fisher’s exact test for categorical variables, as appropriate. Odds ratios (ORs) with 95% confidence intervals (CIs) were calculated, and effect sizes were assessed using Cramér’s V, interpreted as negligible (<0.10), small (0.10–0.29), moderate (0.30–0.49), and large (≥0.50) [24]. Statistical significance was defined as two-sided *p* < 0.05. Missing data were handled using available-case analysis, with percentages calculated using the number of participants with available data for each variable as the denominator. Missing data were not imputed. Given the small extreme-wait cohort and sparse observations across several categories, multivariable modelling was not undertaken. Statistical analyses were performed using MedCalc Statistical Software version 23.2.6 (MedCalc Software Ltd., Ostend, Belgium).

## 3. Results

### 3.1. Study Cohort

**Figure 1:**
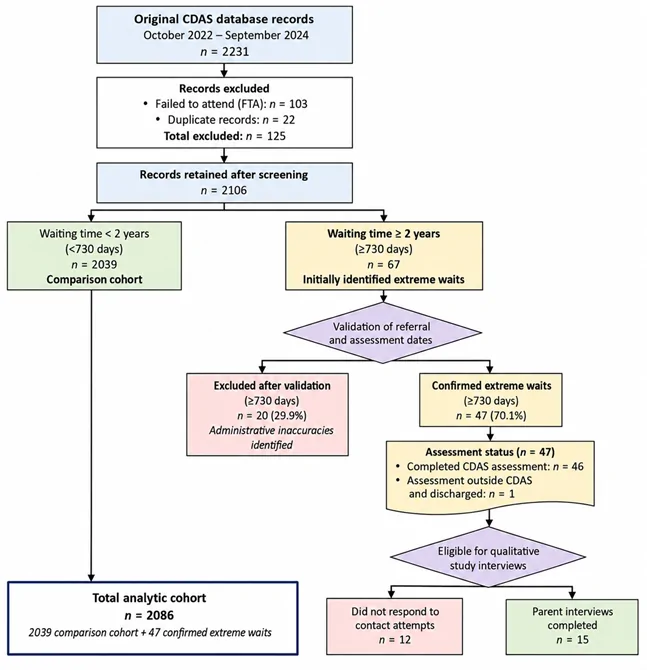
Study cohort derivation and sampling.

Between October 2022 and September 2024, the CDAS database contained 2231 assessment records. Following the exclusion of 103 failed-to-attend records and 22 duplicate records, 2106 records remained for cohort classification: 2039 in the comparison cohort and 67 with an administrative waiting time ≥730 days (Figure 1).

### 3.2. Extreme-Wait Cohort

The extreme-wait cohort underwent manual validation of referral dates, assessment dates and appointment pathways (Figure 1). Twenty children (29.9%) were excluded because an electronic medical record review showed they had not experienced a validated waiting time ≥730 days. These 20 records were excluded from comparative analyses rather than reassigned to the comparison cohort because their administrative waiting-time classifications were affected by identified recording inaccuracies. Administrative inaccuracies included incorrect referral dates and misclassified planned review pathways, leaving 47 children in the validated extreme-wait cohort.

Of these, 46 completed developmental assessment, while one was discharged after assessment outside the service. Analyses of waiting time and age at assessment were therefore based on 46 children, whereas demographic and clinical characteristics were reported for all 47.

The qualitative component included 15 caregivers of children in the validated extreme-wait cohort who completed semi-structured interviews. Twelve families did not respond after two contact attempts. Participant flow is shown in Figure 1.

### 3.3. Validation of Administrative Extreme Waiting-Time Data

Verification of referral and assessment dates identified administrative errors affecting extreme-wait records (Table 2). The most common discrepancies were incorrect referral dates (including use of date of birth), use of original rather than review referral dates for planned reassessments, and inconsistencies in appointment pathway or assessment dates. Following validation, the median waiting time decreased from 873 to 859 days.

### 3.4. Comparison of Children with and Without Extreme Waiting Times

Of the 2086 children included, 47 (2.3%) experienced extreme waiting times (≥730 days), while 2039 comprised the comparison cohort (Table 3). Children in the extreme-wait cohort were more likely to be from culturally and linguistically diverse (CALD) backgrounds (76.6% vs. 53.5%; OR 2.85, 95% CI 1.44–5.63; *p* = 0.002) and to require an interpreter (23.4% vs. 9.0%; OR 3.10, 95% CI 1.55–6.20; *p* = 0.003). No significant differences were observed in sex, Aboriginal and/or Torres Strait Islander status, refugee background, child protection involvement, or the overall distribution of SEIFA IRSD deciles.

Ethnicity differed between cohorts (*p* < 0.001). Children from Middle Eastern backgrounds were more likely to experience extreme waiting times (OR 3.63, 95% CI 1.96–6.73; *p* < 0.001), whereas children of White or Mixed White ethnicity were less likely to do so (OR 0.34, 95% CI 0.16–0.74; *p* = 0.003). No significant differences were observed for other ethnic groups.

Children with extreme waiting times were more often referred by paediatricians (89.4% vs. 74.1%; OR 2.94, 95% CI 1.16–7.46; *p* = 0.017) and were less likely to receive Priority 1 triage but more likely to receive Priority 3 (*p* < 0.001). The median waiting time was 859 days (IQR 775–932) compared with 254 days (IQR 111–355), a difference of 605 days (*p* < 0.001). Children with extreme waits were also more likely to undergo review rather than new assessments (53.2% vs. 21.0%; OR 4.29, 95% CI 2.39–7.69; *p* < 0.001).

### 3.5. Assessment Pathway Characteristics Within the Extreme-Wait Cohort

Among the 47 children with validated extreme waiting times, 22 underwent new assessments, 22 planned review assessments and three unplanned review (Table 4). Median waiting time did not differ between pathways (*p* = 0.23). Age at referral differed, with children undergoing unplanned review assessments referred at older ages. A similar trend was observed for age at assessment but was not statistically significant (*p* = 0.05). Planned review assessments were predominantly Priority 3 referrals and referred by paediatricians, whereas new assessments included more Priority 2 referrals and greater ethnic diversity. The unplanned review subgroup (*n* = 3) was characterised by older referral and assessment ages.

### 3.6. Qualitative Findings

Six overarching themes were identified through thematic analysis and are presented as propositions describing caregivers’ experiences of validated extreme waiting (≥730 days). The qualitative findings are integrated with the quantitative results to explain how families experienced these extreme waits. As interviews were retrospective and purposively sampled, the findings represent caregivers’ reported experiences and perceptions rather than causal relationships or experiences representative of all families.

### 3.7. Extreme Waiting Became an Accepted Expectation

Quantitative analysis demonstrated a median validated waiting time of 859 days. Despite these extreme waits, many caregivers described waiting as an expected feature of publicly funded developmental assessment services, attributing delays to increasing demand, workforce shortages and limited public resources.

*“As you know these specialists’ services are in short supply…”*

*“It’s reasonable because everybody’s waiting.”*

Several caregivers underestimated their documented waiting times despite administrative records confirming waits exceeding two years, suggesting recall bias, normalisation of extreme waiting, or both.

### 3.8. Communication from the Service Shaped Caregivers’ Experiences

The quantitative cohort included a high proportion of CALD children (76.6%), with 23.4% requiring an interpreter. Interviews indicated that communication strongly influenced caregivers’ experiences. Families receiving regular updates generally described greater reassurance despite extreme waits, whereas those reporting extended periods without contact described uncertainty and feeling forgotten. Caregivers consistently recommended regular updates, clearer information about expected waiting times and guidance regarding interim supports.

*“I didn’t hear from them for a whole year…”*

*“Just to say you’re still on our wait list…”*

### 3.9. Extreme Waiting Influenced Perceived Developmental Care Pathways

Although quantitative analysis identified waiting times exceeding two years, caregivers described the effects of extreme waiting as extending beyond healthcare into education, disability and early intervention systems. Many perceived delayed assessment as delaying access to school supports, intervention services and disability funding requiring formal diagnostic documentation. Families receiving ongoing support from paediatricians or allied health professionals generally perceived waiting as more manageable than those without interim supports.

*“The waiting process also delays the child too.”*

*“We actually went on another waiting list while waiting.”*

These findings reflect caregivers’ perceptions and should not be interpreted as evidence that extreme waiting directly caused these reported impacts.

### 3.10. Access to Interim Supports and Service Navigation Influenced Experiences

Quantitative analysis showed that most children experiencing extreme waits were referred by paediatricians. However, interviews suggested that specialist referral alone did not necessarily ensure coordinated support during the waiting period. *“Nobody told us where else we could go.”*

Families with established paediatric, allied health or early intervention services generally described more manageable experiences than those without ongoing supports. Many caregivers reported uncertainty regarding available services and suggested that clearer referral pathways and improved service navigation would have reduced uncertainty during extreme waiting.

*“We didn’t know what services were available while we were waiting.”*

### 3.11. Socioeconomic Disadvantage Limited Alternatives to Public Assessment

Nearly half of the quantitative cohort resided within the most socioeconomically disadvantaged SEIFA decile. Interviews illustrated how financial constraints limited alternatives to public assessment, with many families reporting that private developmental assessments were unaffordable.

*“I don’t really have the finances…”*

*“Our wait was too long.”*

Together, these findings suggest that socioeconomic disadvantage may restrict families perceived ability to access alternative assessment pathways during extreme waiting.

### 3.12. Families Distinguished Dissatisfaction with Waiting from Satisfaction with Clinical Care

Despite dissatisfaction with extreme waiting, most caregivers clearly distinguished the waiting period from the assessment itself. Families generally described the assessment as supportive and reassuring, attributing concerns to system-level delays rather than the quality of clinical care.

*“The assessment itself was amazing.”*

Only one caregiver reported dissatisfaction with the assessment process.

## 4. Mixed-Methods Synthesis

The qualitative findings provided explanatory context for the quantitative analyses. Although the quantitative data identified demographic and clinical characteristics associated with extreme waiting, interviews demonstrated that caregiver experiences were shaped not only by waiting duration but also by communication, service navigation, access to interim supports and socioeconomic circumstances. Together, the findings suggest that improving families’ experiences of extreme waiting requires service improvements extending beyond reducing waiting times alone (Table 5).

## 5. Discussion

### 5.1. Principal Findings

This mixed-methods study provides new insights into validated extreme waiting (≥730 days) for developmental assessment within a publicly funded child developmental assessment service (CDAS). By integrating administrative data validation, quantitative cohort analyses and caregiver interviews, this study demonstrates that extreme waiting is a multifaceted service issue extending beyond waiting duration alone. Manual validation showed that almost one-third of children initially classified as experiencing extreme waiting had been misclassified because of administrative recording inaccuracies or changes in appointment pathways. The validated extreme-wait cohort included a high proportion of CALD families, children requiring interpreters, Priority 3 referrals and planned review appointments, while almost half (48.9%) resided in the most socioeconomically disadvantaged SEIFA IRSD decile. However, the overall distribution of SEIFA IRSD deciles did not differ significantly between the extreme-wait and comparison cohorts. Caregiver interviews complemented these findings by demonstrating that experiences of extreme waiting were influenced not only by waiting duration but also by communication, access to interim supports, service navigation and financial capacity. Together, these findings suggest that evaluation of developmental assessment services should consider data quality, clinical context and caregiver experience alongside conventional waiting-time metrics.

### 5.2. Understanding Validated Extreme Waiting: More than a Measure of Service Capacity

A key finding was that extreme waiting times should not be interpreted as a single measure of service performance. Manual review showed that almost one-third of children initially identified through administrative records as waiting more than two years had been misclassified because the recorded interval did not represent a true extreme wait. Rather than indicating poor database quality, this finding highlights the importance of validating routinely collected administrative data before using waiting-time metrics for service evaluation, benchmarking or resource allocation. Similar concerns have been recognised internationally, where differences in referral pathways, assessment processes and recording practices contribute to variability in reported waiting times [11,12,25].

Validated extreme waiting also represented several distinct clinical pathways rather than a homogeneous cohort. Approximately half of the validated cohort underwent new assessments, while the remainder comprised predominantly planned review appointments. Planned reviews differ fundamentally from initial assessments because reassessment is intentionally scheduled following developmental maturation, therapeutic intervention or transition into formal schooling before diagnostic clarification. Consequently, waiting times for planned reviews should not be interpreted in the same way as delays in accessing an initial developmental assessment. This distinction extends previous research by demonstrating that children experiencing validated extreme waiting do not represent a single group with equivalent clinical needs or service experiences. Instead, interpretation of waiting-time metrics should account for appointment pathway and clinical context, particularly when planned review appointments are included within service waiting lists [10,11,12].

Children experiencing validated extreme waiting also differed from the remaining CDAS cohort. CALD families, interpreter users and children triaged as Priority 3 were disproportionately represented, while almost half resided within the most socioeconomically disadvantaged SEIFA decile. These findings should be interpreted cautiously because this observational study identified associations within a single service rather than causal determinants of extreme waiting. Furthermore, comparisons were made with the remaining CDAS cohort rather than a prospectively recruited cohort of children experiencing shorter waiting times. Accordingly, these characteristics describe the profile of children represented within the validated extreme-wait cohort rather than predictors of extreme waiting.

The prominence of CALD families is consistent with previous work from the same health district, demonstrating inequities in access to developmental assessment [6]. Similarly, Teoh et al. [13] and Smith-Young et al. [9] identified language and communication barriers and difficulties navigating health services as important influences on caregiver experiences during developmental assessment pathways. Collectively, these findings suggest that equitable developmental assessment extends beyond timely access and includes culturally responsive communication, service navigation and support while families await assessment.

Socioeconomic disadvantage also warrants careful interpretation. Although almost half of the validated extreme-wait cohort resided within the most disadvantaged SEIFA decile, socioeconomic disadvantage as an independently adjusted association with extreme waiting was not estimated in comparison analyses. The qualitative findings provide important context by showing that financial circumstances influenced caregivers’ options once extreme waiting had already occurred. Families frequently described private assessment as unaffordable, leaving them dependent on publicly funded services despite extended delays. Thus, socioeconomic disadvantage did not differ significantly between the extreme-wait and comparison cohorts but may substantially influence families’ capacity to circumvent or mitigate its consequences. This distinction has received limited attention in previous developmental assessment research and warrants further investigation.

### 5.3. Understanding Caregivers’ Experiences of Validated Extreme Waiting

Although children in the quantitative cohort experienced a median validated waiting time of 859 days, caregiver interviews demonstrated that waiting duration alone did not determine how families experienced extreme waiting. Rather, communication, service navigation, access to interim supports and financial capacity consistently shaped caregiver experiences. These findings complement the quantitative analyses by demonstrating that families experiencing similar extreme waits did not necessarily report the same experiences, further highlighting the limitations of evaluating developmental assessment services using waiting-time metrics alone.

Many caregivers described extreme waiting as an anticipated feature of accessing publicly funded developmental assessment services rather than an unexpected event. They commonly attributed delays to increasing demand, workforce shortages and broader pressures within the public health system, consistent with international studies reporting that families adapt their expectations while navigating lengthy diagnostic pathways and multiple waiting lists [26]. However, several caregivers substantially underestimated their documented waiting times. As interviews were retrospective, this discrepancy may reflect recall bias, normalisation of extreme waiting, or both. Consequently, caregiver acceptance of extreme waiting should not be interpreted as evidence that such waiting times are clinically acceptable or without consequence.

### 5.4. Communication Emerged as One of the Strongest Influences on Caregiver Experience

Families consistently described regular updates, reassurance that referrals remained active and clear information regarding expected waiting times as reducing uncertainty during extreme waits. Conversely, extreme periods without contact often left caregivers feeling forgotten and uncertain about available supports. Although these findings reflect caregiver perceptions and were not independently verified against service records, they are consistent with previous Australian research demonstrating that caregiver satisfaction is strongly associated with communication, clinician engagement and information provision despite extended waiting periods [13]. Similarly, Smith-Young et al.’s systematic review identified communication, continuity of care and difficulties navigating diagnostic pathways as recurring themes across international qualitative studies [9]. Together, these findings suggest that communication represents a potentially modifiable aspect of service delivery independent of waiting duration.

Caregivers also described extreme waiting as extending beyond healthcare into education, disability and early intervention systems. Rather than attributing developmental outcomes directly to extreme waiting, families frequently reported difficulties accessing educational supports, disability funding and intervention services requiring formal diagnostic documentation. Similar observations have been reported internationally, where developmental assessment delays were experienced as a series of interconnected barriers rather than a single delay [26]. Accordingly, these findings should be interpreted as caregivers’ perceptions of how extreme waiting influenced their experiences rather than evidence of causal effects on developmental or educational outcomes.

An important mixed-methods finding was that families with similar waiting durations often described markedly different experiences. Although almost 90% of children experiencing extreme waiting had been referred by paediatricians, interviews suggested that specialist referral alone did not ensure coordinated support throughout the waiting period. Families receiving ongoing support from paediatricians, allied health professionals or early intervention providers generally described waiting as more manageable than those without established supports. These findings complement previous Australian studies demonstrating longer waiting times within public developmental assessment services than private pathways [7,10] and broader qualitative evidence that financial resources influence families’ ability to navigate complex diagnostic systems [27]. Recent service innovations, including the LANTERN triage model and coordinated developmental assessment pathways, similarly emphasise supporting families throughout the diagnostic journey rather than focusing exclusively on reducing waiting times [28]. Although this study cannot determine whether interim supports improve developmental outcomes, caregivers consistently perceived them as making extreme waiting more manageable.

Finally, caregivers consistently distinguished dissatisfaction with extreme waiting from satisfaction with the developmental assessment itself. Most participants described the multidisciplinary assessment as comprehensive, supportive and reassuring despite frustration with the preceding wait. Similar findings were reported by Teoh et al., who observed high caregiver satisfaction with clinician communication and assessment quality despite concerns regarding waiting times [13]. This distinction suggests that opportunities for service improvement relate primarily to the period before assessment, particularly communication, service navigation and access to interim supports, rather than to the quality of the assessment itself.

### 5.5. Comparison with Previous Literature

The present study extends previous Australian and international research in several important ways [2,4,10,11]. Previous studies have primarily focused on reducing waiting times through service redesign, triage innovations and alternative models of care [5,8,28,29,30,31], whereas comparatively few have examined children experiencing validated extreme waiting or integrated caregiver experiences with quantitative service data.

First, manual validation demonstrated that almost one-third of children initially classified as experiencing extreme waiting had been misclassified, highlighting the importance of validating administrative datasets before interpreting service performance or benchmarking waiting times.

Second, comparison with the remaining CDAS cohort identified characteristics overrepresented among children experiencing validated extreme waiting, including CALD families, Priority 3 referrals and planned review appointments. These findings describe the profile of this cohort rather than causal determinants of extreme waiting.

Third, the mixed-methods design provided a more comprehensive understanding of validated extreme waiting by integrating quantitative analyses with caregiver experiences. Whereas quantitative analyses identified which children experienced extreme waiting, qualitative findings explained why families with similar waiting durations described markedly different experiences.

The findings also extend previous work from the same CDAS. Earlier studies demonstrated geographical and sociodemographic inequities in access to developmental assessment [6], diagnostic delays within publicly funded services [7], improved access through targeted School Starter Blitz clinics [8], and generally positive caregiver experiences despite routine waiting periods [13]. The present study complements this work by demonstrating that validated extreme waiting reflects a combination of administrative processes, clinical pathways and caregiver experiences rather than service capacity alone.

### 5.6. Clinical and Service Implications

The findings have several implications for developmental assessment services. First, routine validation of administrative data should be incorporated into service evaluation before waiting-time metrics are interpreted or used for benchmarking. Although this finding relates specifically to the extreme-wait cohort, it demonstrates that recording inaccuracies and changes in appointment pathways can substantially affect reported waiting times and service performance.

Second, extreme waiting should not be reported as a single performance indicator. Almost half of the validated extreme-wait cohort comprised planned review appointments that reflected intentional clinical decision making rather than delayed access to an initial assessment. Distinguishing planned review from initial assessment pathways would provide a more meaningful evaluation of service performance.

Third, caregiver interviews identified communication, service navigation and access to interim supports as modifiable aspects of the waiting experience. Supporting families during extreme waiting should therefore complement efforts to reduce waiting times. Family-navigation programmes have shown promise in improving engagement with diagnostic pathways and facilitating earlier access to intervention services, particularly for families experiencing barriers to care [27]. Although the present study was not designed to evaluate these interventions, caregivers consistently perceived regular communication and coordinated support as valuable during extreme waiting.

Finally, the overrepresentation of CALD families and interpreter users within the validated extreme-wait cohort, together with the high proportion of children residing in the most socioeconomically disadvantaged areas, highlights the importance of equity-focused service planning. Although CALD status and interpreter requirement should not be interpreted as causal determinants of extreme waiting, and the overall SEIFA IRSD distribution did not differ significantly between cohorts, these findings identify important considerations for culturally responsive communication, proactive follow-up and support with navigation of developmental, educational and disability services.

## 6. Strengths and Limitations

This study has several strengths. To our knowledge, it is one of the first Australian mixed-methods studies of validated extreme waiting for developmental assessment, combining administrative data validation and quantitative comparison with the remaining CDAS cohort and qualitative caregiver interviews. This approach provided a more comprehensive understanding of extreme waiting than either method alone.

Several limitations should be acknowledged. First, this single-service study was conducted within a culturally diverse metropolitan health district, limiting generalisability to services with different populations, referral pathways or models of care. Second, the relatively small extreme-wait cohort and sparse observations across several categories limited statistical power and precluded multivariable modelling; the observed associations should therefore not be interpreted as causal. Interviews were conducted retrospectively and may have been affected by recall bias, particularly regarding waiting duration. Although the quantitative cohort included many CALD families and children requiring interpreters, only one interview used an interpreter, so the experiences of families with limited English proficiency may not have been fully captured. In total, 12 of the 27 approached families (44.4%) did not participate despite two contact attempts, introducing potential non-response bias. Although the interviewer was not involved in the clinical care of the participants, one investigator worked within the CDAS; reflexive practice, independent coding and consensus discussions were used to minimise potential bias. The waiting times of the comparison cohort were not validated like the extreme waiting times cohort for this work due to logistic issues; however, internal processes of data entry and checks have been made more robust within our service, creating confidence in the data. Finally, although quantitative and qualitative findings were integrated through convergent interpretation within the embedded mixed-methods design, integration occurred primarily at the study rather than participant level. Consequently, this study could not determine whether caregiver experiences differed according to demographic or clinical characteristics. Future mixed-methods studies incorporating participant-level integration may provide a more comprehensive understanding of factors influencing experiences of extreme waiting.

## 7. Conclusions

Validated extreme waiting for developmental assessment represents more than a measure of service capacity. This study demonstrates that extreme waiting reflects a combination of genuine delays, clinically planned review pathways and administrative recording inaccuracies, while caregiver experiences are shaped by communication, service navigation, access to interim supports and financial capacity. Together, these findings suggest that evaluation of developmental assessment services should extend beyond waiting-time metrics to consider data quality, clinical context, equity and caregiver experience.

Routine validation of administrative data, differentiation of planned review pathways from initial assessment delays, culturally responsive communication and improved navigation to interim supports represent practical opportunities to strengthen developmental assessment services during extreme waiting. Future research should prospectively evaluate these strategies and examine whether they improve caregiver experience, equity of access and child outcomes.

## Figures and Tables

**Table 1:** Child developmental assessment service locations and selected suburb-level indicators.

Suburb	Population (2021)	% <14 yrs	SEIFA IRSAD * (2021)	AEDC DV1 * (2020/21)	AEDC DV1 (2024)	AEDC DV2 * (2020/21)	AEDC DV2 (2024)
**Bankstown**	186,245	21.1%	3	27.4%	28.1%	13.9%	14.6%
**Fairfield**	208,475	17.9%	2	31.8%	32.4%	17.2%	17.8%
**Campbelltown**	8007	22.8%	2	29.9%	30.7%	15.2%	15.9%
**Liverpool**	233,446	22.1%	4	24.6%	25.3%	11.5%	12.1%
**Bowral**	13,626	14.2%	8	14.9%	15.6%	5.8%	6.3%

* Suburb-level indicators: socioeconomic status, developmental vulnerability and demographics. DV1: % developmentally vulnerable on ≥1 domain; DV2: % developmentally vulnerable on ≥2 domains on Australian Early Development Census (AEDC). SEIFA IRSAD: Socio-Economic Indexes for Areas Index of Relative Socio-Economic Advantage and Disadvantage; decile 1 indicates relatively greater disadvantage and lack of advantage, while decile 10 indicates relatively greater advantage and lack of disadvantage. Based on AEDC community profiles and ABS SEIFA.

**Table 2:** Validation of administrative records for children initially identified as having extreme waiting times (≥730 days).

Administrative Validation	Overall (*n* = 67) ‡	New (*n* = 30) ‡	Planned Review (*n* = 27) ‡	Unplanned Review (*n* = 10) ‡	*p* §
**Validation outcome**					
Excluded (<730 days)	20 (29.9)	8 (26.7)	5 (18.5)	7 (70.0)	0.011
Confirmed ≥730 days	47 (70.1)	22 (73.3)	22 (81.5)	3 (30.0)	
**Assessment outcome**					
Completed assessment	46	22	21	3	–
Assessed elsewhere and discharged †	1	0	1	0	–

Data are presented as *n* (%). † One child underwent developmental assessment through another service and was subsequently discharged from the CDAS waiting list without receiving a CDAS assessment. ‡ Percentages for assessment pathways are column percentages. § Fisher’s exact test compares validation outcomes across assessment pathways.

**Table 3:** Comparison between the CDAS comparison cohort and children experiencing extreme waiting times (≥730 days).

Characteristic	Comparison Cohort (*n* = 2039)	Extreme-Wait Cohort (*n* = 47)	Odds Ratio or Effect Size (95% CI)	*p* Value
Male sex †	1396/1977 (70.6%)	30/47 (63.8%)	0.73 (0.40–1.34)	0.333
Aboriginal and/or Torres Strait Islander ‡	131/1836 (7.1%)	1/47 (2.1%)	0.28 (0.04–2.07)	0.252
**CALD background †**	**998/1866 (53.5%)**	**36/47 (76.6%)**	**2.85 (1.44–5.63)**	**0.002**
Refugee/asylum-seeker background ‡	36/1997 (1.8%)	1/47 (2.1%)	1.18 (0.16–8.82)	0.580
Known to child protection services ‡	58/1997 (2.9%)	1/47 (2.1%)	0.73 (0.10–5.36)	1.000
**Interpreter required †**	**179/1997 (9.0%)**	**11/47 (23.4%)**	**3.10 (1.55–6.20)**	**0.003**
**Ethnicity §**			**Effect size = 0.120**	**<0.001**
**Middle Eastern †**	**254/2039 (12.5%)**	**16/47 (34.0%)**	**3.63 (1.96–6.73)**	**<0.001**
Asian †	495/2039 (24.3%)	16/47 (34.0%)	1.61 (0.87–2.97)	0.125
**White and Mixed White †**	**764/2039 (37.5%)**	**8/47 (17.0%)**	**0.34 (0.16–0.74)**	**0.003**
Pacific Islander ‡	54/2039 (2.6%)	3/47 (6.4%)	2.51 (0.75–8.32)	0.135
Other ‡	191/2039 (9.4%)	2/47 (4.3%)	0.43 (0.10–1.79)	0.312
Unknown/not recorded ‡	281/2039 (13.8%)	2/47 (4.3%)	0.28 (0.07–1.15)	0.080
**National SEIFA IRSD decile §**			**Effect size = 0.077**	0.121
Decile 1 †	537/1391 (38.6%)	23/47 (48.9%)	1.52 (0.85–2.73)	0.172
Decile 2 ‡	222/1391 (16.0%)	3/47 (6.4%)	0.36 (0.11–1.17)	0.099
Decile 3 ‡	48/1391 (3.5%)	1/47 (2.1%)	0.61 (0.08–4.50)	1.000
Decile 4 ‡	97/1391 (7.0%)	1/47 (2.1%)	0.29 (0.04–2.13)	0.368
Decile 5 †	69/1391 (5.0%)	5/47 (10.6%)	2.28 (0.87–5.95)	0.089
Deciles 6–10 †	418/1391 (30.1%)	14/47 (29.8%)	0.99 (0.52–1.86)	1.000
**Referrer †**			**Effect size = 0.052**	**0.018**
**Paediatrician †**	**1511/2039 (74.1%)**	**42/47 (89.4%)**	**2.94 (1.16–7.46)**	**0.017**
**Allied health/other †**	**528/2039 (25.9%)**	**5/47 (10.6%)**	**0.34 (0.13–0.87)**	**0.017**
**Priority at triage †**			**Effect size = 0.094**	**<0.001**
**Priority 1 ‡**	**469/1713 (27.4%)**	**2/47 (4.3%)**	**0.12 (0.03–0.49)**	**<0.001**
Priority 2 †	563/1713 (32.9%)	15/47 (31.9%)	0.96 (0.51–1.78)	1.000
**Priority 3 †**	**681/1713 (39.8%)**	**30/47 (63.8%)**	**2.67 (1.46–4.89)**	**0.001**
Median wait time, days *	254 (111–355)	859 (775–932)	Median difference, 605 days	**<0.001**
**Assessment type †**			**Effect size = 0.121**	**<0.001**
New	1475/1866 (79.0%)	22/47 (46.8%)	**OR 0.23 (0.13–0.42)**	**<0.001**
Review	391/1866 (21.0%)	25/47 (53.2%)	**OR 4.29 (2.39–7.69)**	**<0.001**

Data are *n*/N (%) or median (IQR). Odds ratios compare each category with all remaining participants. * Mann–Whitney U test. Waiting-time analysis included participants with available waiting-time data (comparison cohort, *n* = 2037; extreme-wait cohort, *n* = 46). † Pearson’s chi-square test. ‡ Fisher’s exact test. § Monte Carlo Fisher exact test for sparse multi-category tables. Overall effect size for multi-category variables is calculated for chi square using Cramér’s V. Cramér’s V was interpreted as negligible (<0.10), small (0.10–0.29), moderate (0.30–0.49), and large (≥0.50), and statistically significant results (*p* < 0.05) are shown in bold. CALD, culturally and linguistically diverse; SEIFA IRSD, Socio-Economic Indexes for Areas Index of Relative Socio-Economic Disadvantage.

**Table 4:** Demographic and clinical characteristics of children with validated extreme waiting times (≥730 days), stratified by assessment pathway.

Characteristic	Overall (*n =* 47)	New Assessment (*n =* 22)	Planned Review (*n =* 22)	Unplanned Review (*n =* 3)	*p* Value
**Demographic characteristics**					
Male sex	30 (63.8)	16 (72.7)	11 (50.0)	3 (100.0)	—
**Age and waiting time**					
Waiting time, days *	859 (775–932)	819 (793–969)	890 (821–924)	762 (738–767)	0.23
Age at referral, months	33 (23–34.7)	29 (25–34)	34 (29–37)	83 (70–83)	**0.02**
Age at assessment, months *	62 (57–66)	59 (53–62)	63 (57.8–65)	108 (94–108)	0.05
**Vulnerability characteristics**					
Aboriginal and/or Torres Strait Islander	1 (2.1)	1 (4.5)	0	0	—
CALD background	36 (76.6)	13 (59.1)	20 (90.9)	3 (100.0)	—
Refugee or asylum-seeker background	1 (2.1)	0	1 (4.5)	0	—
Known to child protection services	1 (2.1)	1 (4.5)	0	0	—
Interpreter required	11 (23.4)	3 (13.6)	7 (31.8)	1 (33.3)	—
**Ethnicity**					
Middle Eastern	16 (34.0)	5 (22.7)	9 (40.9)	2 (66.7)	
Asian	16 (34.0)	6 (27.3)	9 (40.9)	1 (33.3)	
White or Mixed White	8 (17.0)	7 (31.8)	1 (4.5)	0	
Pacific Islander	3 (6.4)	2 (9.1)	1 (4.5)	0	
Other	2 (4.3)	1 (4.5)	1 (4.5)	0	
Unknown/not recorded	2 (4.3)	1 (4.5)	1 (4.5)	0	
**Socioeconomic characteristics**					
SEIFA IRSD decile 1	23 (48.9)	12 (54.5)	8 (36.4)	3 (100.0)	
SEIFA IRSD decile 2	3 (6.4)	2 (9.1)	1 (4.5)	0	
SEIFA IRSD decile 3	1 (2.1)	1 (4.5)	0	0	
SEIFA IRSD decile 4	1 (2.1)	0	1 (4.5)	0	
SEIFA IRSD decile 5	5 (10.6)	2 (9.1)	3 (13.6)	0	
SEIFA IRSD deciles 6–10	14 (29.8)	5 (22.7)	9 (40.9)	0	
**Referral characteristics**					
Paediatrician	42 (89.4)	17 (77.3)	22 (100.0)	3 (100.0)	
Allied health or other	5 (10.6)	5 (22.7)	0	0	
**Clinical characteristics**					
Priority 1	2 (4.3)	2 (9.1)	0	0	
Priority 2	15 (31.9)	11 (50.0)	2 (9.1)	2 (66.7)	
Priority 3	30 (63.8)	9 (40.9)	20 (90.9)	1 (33.3)	

Data are presented as *n* (%) for categorical variables and median (IQR) for continuous variables. * Waiting time and age at assessment were available for 46 children who completed CDAS assessment. Percentages in the overall column were calculated using the total number of children with validated extreme waiting times. Percentages in the new assessment, planned review, and unplanned review columns were calculated using the number of children within each assessment pathway (*n* = 22, *n* = 22 and *n* = 3, respectively). *p*-values for continuous variables were calculated using the Kruskal–Wallis test.

**Table 5:** Integrated quantitative and qualitative findings with representative caregiver quotations.

Integrated Mixed-Methods Finding	Quantitative Finding	Qualitative Interpretation	Representative Caregiver Quotations
**1. Extreme waiting became an accepted expectation for many families**	Median validated waiting time was **859 days** (IQR 775–932), substantially exceeding intended triage timeframes.	Many caregivers perceived extreme waiting (≥730 days) as an anticipated feature of accessing public developmental assessment services. Several underestimated their documented waiting times, suggesting recall bias, normalisation of extreme waiting, or both.	• *“As you know these specialist services are in short supply, so yes, we have to wait.”* • *“It’s reasonable because everybody’s waiting.”* • *“There’s a lot of neurodivergent children coming through…there’s a lot of families that don’t have the finances to pay for a private assessment.”*
**2. Communication shaped caregivers’ experiences of extreme waiting**	**76.6%** of children were from CALD backgrounds, and **23.4%** required an interpreter.	Caregivers consistently reported that communication influenced how they experienced extreme waiting. Families valued reassurance, regular updates and transparency regarding waiting times more than frequent contact alone.	• *“I didn’t hear from them for a whole year until the appointment came up.”* • *“Just to say you’re still on our wait list.”* • *“It would be better if you got updates because you didn’t feel lost.”*
**3. Extreme waiting was perceived to influence children’s developmental care pathways**	Children experienced validated waits exceeding **2 years** before assessment.	Caregivers perceived extreme waiting as extending beyond healthcare into education, disability and early intervention systems, delaying access to services requiring formal assessment or diagnosis. These represent caregiver perceptions rather than demonstrated causal effects.	• *“The waiting process also delays the child too.”* • *“We actually went on another waiting list while we were waiting.”* • *“They wouldn’t be able to start until they have that paperwork.”*
**4. Access to interim supports and service navigation influenced experiences of extreme waiting**	**89.4%** of referrals originated from paediatricians, although experiences differed despite similar waiting times.	Families with ongoing paediatric, allied health or early intervention support generally perceived extreme waiting as more manageable. Caregivers frequently reported difficulties navigating available services while awaiting assessment.	• *“He was already getting all the therapy before he even got the diagnosis.”* • *“No daycare, no early intervention, nothing because I had to wait.”* • *“They should kind of direct me to where I can go.”* • *“I didn’t even know there was an early intervention school.”*
**5. Socioeconomic disadvantage limited alternatives to public assessment**	**48.9%** of children resided in SEIFA IRSD Decile 1 (greatest disadvantage).	Caregivers described financial circumstances as influencing whether private assessment was feasible. Families with fewer financial resources perceived themselves as having little alternative to remaining on public waiting lists.	• *“I don’t really have the finances to pay for a private assessment.”* • *“Our wait was too long.”* • *“We have to pay a lot of money.”*
**6. Families distinguished dissatisfaction with extreme waiting from satisfaction with clinical care**	Despite extreme waiting, almost all children ultimately completed assessment within CDAS.	Most caregivers differentiated dissatisfaction with system-level waiting from satisfaction with the quality of the assessment and clinical staff once assessment occurred.	

## Data Availability

All de-identified is shared in the Supplementary Materials File.

## Supplementary Materials

The following supporting information can be downloaded at https://www.mdpi.com/article/10.3390/children13081088/s1. Deidentified Data files, and Child Developmental Assessment Service (CDAS) Model of Care.

## Appendix A. Good Reporting of a Mixed Methods Study (GRAMMS) Reporting Guidelines Procedure

**GRAMMS Criterion**	**What Should Be Reported**	**Report in Your Study**
**1. Rationale for using mixed methods**	Clear explanation of why both quantitative and qualitative methods are needed and how they complement each other.	Mixed methods enabled examination of both objective factors contributing to extreme waits and caregiver experiences of those waits.
**2. Description of mixed-methods design**	Specify design type (convergent, explanatory sequential, exploratory sequential, embedded) and provide justification.	Embedded mixed-methods design combining retrospective quantitative data review with qualitative caregiver interviews.
**3. Timing of data collection**	Explain whether data were collected concurrently or sequentially.	Quantitative data were extracted and validated prior to qualitative sampling. Statistical analysis and caregiver interviews were subsequently conducted concurrently.
**4. Priority of methods**	State whether qualitative or quantitative strand has equal or greater emphasis.	Quantitative-dominant design with a complementary qualitative component.
**5. Sampling strategy for each strand**	Describe sampling approach and relationship between quantitative and qualitative samples.	Quantitative: all children with recorded waits ≥730 days (730 days) compared with rest of CDAS data cohort. Qualitative: purposive maximum-variation sampling of caregivers from the validated extreme-wait cohort and compared with the rest of the cohort.
**6. Data collection methods**	Clearly describe tools/instruments/interviews used for both strands.	Quantitative data obtained from the CDAS database and eMR review. Qualitative data collected through semi-structured caregiver telephone interviews.
**7. Data analysis procedures**	Explain analytical methods used for both quantitative and qualitative data.	Descriptive and comparative statistical analyses were performed. Interview transcripts underwent thematic analysis using Braun and Clarke methodology.
**8. Integration of data**	Describe how data were merged, connected, or embedded (e.g., joint displays, comparison, narrative integration).	Quantitative and qualitative findings were integrated through narrative comparison and interpretation in the results and discussion. Embedded mixed-methods design with quantitative dominance and convergent interpretation.
**9. Interpretation of integrated findings**	Explain how combined findings were interpreted to generate meta-inferences.	Combined findings demonstrated that extreme waits reflected administrative inaccuracies, planned review pathways and genuine service delays, while highlighting caregiver experiences during waiting periods.
**10. Limitations of mixed-methods design**	Discuss challenges of integrating methods, such as timing, sampling differences, or interpretation issues.	Qualitative findings may not represent all families, and retrospective data collection may have introduced recall bias and limited integration across datasets.

## Appendix B. CDAS Model of Care

See Supplementary Materials Appendix S.

## Appendix C. Text Message and Interview Scripts

1. A text message will be sent to the patient’s emergency contact stating:
  “Hello [insert emergency contact’s name], the SWSLHD CDAS team attempted to contact you today to hear your feedback about the assessment [insert child’s name] had in [insert month year]. We will make another attempt to speak to you [insert time frame e.g., 1–2 days] from a private number. We would like to hear about your experience. SWSLHD CDAS team”
2. Two call attempts to contact the patient’s emergency contact will be made on a single day.
  - If contact is not made by the second call, a follow-up text message will be sent stating: “Hello [*insert emergency contact’s name*], the SWSLHD CDAS team attempted to contact you today to attempted to contact you today to hear your feedback about the assessment [*insert child’s name*] had in [*insert month year*]. We will make another attempt to speak to you [*insert time frame* e.g., *1–2 days*] from a private number. We would like to hear your feedback. SWSLHD CDAS team.”
  - If contact is not made on the second day attempted, no further attempts will be made to contact this family.
3. Upon successful contact with the family, the following script sequence will be used:
  a. The purpose of the call will be explained. “[*Child’s name*] had a child development assessment with our service in [*month year*]. We are analysing the results of our wait times and would like to hear about your experience.”
  b. Verbal consent to participate in the feedback experience will be obtained.
    - Call will be terminated if consent not given.
  c. Permission will be asked to audio-record the conversation, for ease of transcription. If family does not want the conversation recorded, only written notes will be kept.
  d. “Do you recall how long your wait time for [*child’s name*]’s assessment with the CDAS team was?”
  e. “What was your wait time experience like for [*child’s name*]’s assessment with the CDAS team?”
  f. “Do you recall any factors that may have contributed to the waiting time you experienced?”
    - “Were you offered any earlier appointments but unable to attend for any reason?”
    - “Were there any gaps in communication (such as not receiving an appointment letter or confusion regarding appointment date)?”
  g. “Were you linked with intervention services or programs whilst on the waitlist?”
  h. “Was the appointment a new or review assessment?”
  i. “Why do you think the appointment took so long?”
  j. “Do you have any other comments or questions?”
  k. The contact will be thanked for their time and call terminated.

## Appendix D. Consolidated Criteria for Reporting Qualitative Research (COREQ) Checklist

**No.**	**Item**	**Guide Question/Description**	**Reported in This Study**	**Location in Manuscript**
**Domain 1: Research team and reflexivity**				
1	Interviewer/facilitator	Which author(s) conducted the interview?	Semi-structured interviews were conducted by the first author.	Methods—Qualitative Data, p. 6
2	Credentials	What were the researcher’s credentials?	Authors involved in the qualitative component held PhD and Bachelor of Medicine qualifications.	Author information/Methods
3	Occupation	What was their occupation at the time of the study?	Research/clinical investigators undertaking a child developmental assessment service evaluation. The first author had no prior involvement with the CDAS; one investigator had a clinical role within CDAS.	Methods—Qualitative Data/Analysis, p. 6; Strengths and Limitations, p. 17
4	Gender	Was the researcher male or female?	The interviewer was female.	Methods—Qualitative Data Analysis, p. 6
5	Experience and training	What experience or training did the researcher have?	Qualitative analysis was undertaken by the first two authors using Braun and Clarke’s six-step thematic analysis framework, with independent coding, consensus resolution and researcher reflexivity. Specific formal qualitative-research training or years of experience were not reported.	Methods—Qualitative Data Analysis, p. 6
6	Relationship established	Was a relationship established prior to study commencement?	The first author conducting the interviews had no prior involvement with the CDAS or the clinical care of most participants.	Methods—Qualitative Data, p. 6
7	Participant knowledge of interviewer	What did participants know about the researcher?	Families were informed that the CDAS team was analysing waiting times and wished to hear about their experience of the assessment and waiting period. The purpose of the call was explained before consent.	Appendix C, pp. 21–22
8	Interviewer characteristics	What characteristics/biases were reported?	Researcher reflexivity was maintained throughout analysis. One investigator’s clinical role within CDAS was acknowledged, and independent coding, collaborative interpretation and consensus discussions were used to minimise potential bias.	Methods—Qualitative Data Analysis, p. 6; Strengths and Limitations, p. 17
**Domain 2: Study design**				
9	Methodological orientation and theory	What methodological orientation underpinned this study?	Interview transcripts were analysed using Braun and Clarke’s six-step thematic analysis framework: familiarisation, initial coding, searching for themes, reviewing themes, defining themes and reporting findings.	Methods—Qualitative Data Analysis, p. 6
10	Sampling	How were participants selected?	Purposive maximum-variation sampling was used to capture diverse experiences across assessment pathway, local government area, cultural background, child age and waiting time.	Methods—Qualitative Data, p. 6
11	Method of approach	How were participants approached?	Eligible families were approached by text message followed by telephone contact, according to a predefined contact procedure.	Methods—Qualitative Data, p. 6; Appendix C, pp. 21–22
12	Sample size	How many participants were in the study?	15 caregivers completed semi-structured interviews.	Methods—Qualitative Data, p. 6; Results—Study Cohort, p. 17
13	Non-participation	How many refused/dropped out and why?	27 families were approached; 15 participated and 12 did not respond after two contact attempts.	Methods—Qualitative Data, p. 6; Results—Study Cohort, p. 8; Strengths and Limitations, p. 17
14	Setting of data collection	Where were data collected?	Interviews were conducted by telephone.	Methods—Qualitative Data, p. 6
15	Presence of non-participants	Was anyone else present?	No other person was present during the interviews.	Methods—Qualitative Data Analysis, p. 6
16	Description of sample	What were the important participant characteristics?	Participants were parents or primary caregivers of children with validated extreme waiting times (≥730 days). Maximum-variation sampling incorporated assessment pathway, geographic area, cultural background, child age and waiting time.	Methods—Qualitative Data, p. 6
17	Interview guide	Were questions/prompts provided or pilot tested?	Interviews followed a semi-structured interview schedule. The contact procedure, interview questions and prompts are reproduced in Appendix C.	Methods—Qualitative Data, p. 6; Appendix C, pp. 21–22
18	Repeat interviews	Were repeat interviews carried out?	No repeat interviews were conducted.	Methods—Qualitative Data Analysis, p. 6
19	Audio/visual recording	Was audio or visual recording used?	Interviews were audio-recorded with consent. If a family declined recording, written notes were retained.	Methods—Qualitative Data, p. 6; Appendix C, pp. 21–22
20	Field notes	Were field notes made?	Written notes were used where audio recording was declined. Routine additional field-note collection was not reported.	Appendix C, pp. 21–22
21	Duration	What was the duration of interviews?	Interviews lasted approximately 10–15 min.	Methods—Qualitative Data, p. 6
22	Data saturation	Was data saturation discussed?	Yes. Recruitment ceased once thematic saturation was achieved.	Methods—Qualitative Data Analysis, p. 6
23	Transcripts returned	Were transcripts returned for comment/correction?	Transcripts were not routinely returned to all participants for comment or correction.	Methods—Qualitative Data Analysis, p. 6
**Domain 3: Analysis and findings**				
24	Number of data coders	How many researchers coded the data?	Transcripts were coded independently by the first two authors, with discrepancies resolved by consensus.	Methods—Qualitative Data Analysis, p. 6
25	Description of coding tree	Was a coding tree provided?	Analysis followed Braun and Clarke’s six-step framework, progressing from initial coding through development, review and definition of themes. A separate formal coding tree was not presented.	Methods—Qualitative Data Analysis, p. 6
26	Derivation of themes	Were themes identified in advance or derived from data?	Themes were developed through thematic analysis of the interview transcripts using Braun and Clarke’s six-step framework. Six overarching themes were identified.	Methods—Qualitative Data Analysis, pp. 6–7; Results—Qualitative Findings, pp. 11–12
27	Software	What software was used to manage the data?	No dedicated qualitative-analysis software was used; coding was undertaken manually.	Methods—Qualitative Data Analysis, p. 6
28	Participant checking	Did participants provide feedback on findings?	Member checking was not undertaken for all participants.	Methods—Qualitative Data Analysis, p. 6
29	Quotations presented	Were participant quotations used to illustrate findings?	Yes. Representative caregiver quotations are presented to illustrate the identified themes.	Results—Qualitative Findings, pp. 11–12; Table 5, pp. 13–14
30	Data and findings consistent	Was there consistency between data and findings?	Yes. Representative caregiver quotations support the themes and qualitative interpretations reported.	Results—Qualitative Findings, pp. 11–12; Table 5, pp. 13–14
31	Clarity of major themes	Were major themes clearly presented?	Yes. Six overarching themes describing caregiver experiences of extreme waiting are clearly presented.	Results—Qualitative Findings, pp. 11–12; Table 5, pp. 13–14
32	Clarity of minor themes/diverse cases	Were diverse or minor cases discussed?	Yes. Divergent experiences were reported, including differences associated with access to interim supports and communication, and the distinction between dissatisfaction with waiting and satisfaction with the eventual assessment.	Results—Qualitative Findings, pp. 11–12; Table 5, pp. 13–14
